# Prevalence and correlates of disability among urban–rural older adults in Southwest China: a large, population-based study

**DOI:** 10.1186/s12877-022-03193-2

**Published:** 2022-06-24

**Authors:** Runjuan Qiao, Shuli Jia, Wanyu Zhao, Xin Xia, Qiaoli Su, Lisha Hou, Daiping Li, Fengjuan Hu, Birong Dong

**Affiliations:** 1 National Clinical Research Center for Geriatrics and Department of General Practice, West China Hospital, Sichuan University, Chengdu, China; 2 Center of Gerontology and Geriatrics, West China Hospital, Sichuan University, Chengdu, China

**Keywords:** disability, Older adults, Urban–rural, ADL, Barthel index, Correlates

## Abstract

**Background:**

As one of the challenges of aging, older adults with disabilities are often overlooked in remote areas of many developing countries, including southwest China. Similar populations would undoubtedly benefit from a representative, high-quality survey of large samples, which would also enrich global disability data. This study aims to assess the prevalence of disability and associated factors among urban and rural older adults in a typical representative region.

**Method:**

A large-scale baseline survey was conducted between March and September 2020 using face-to-face interviews with a multistage stratified random sample of 16,536 participants aged ≥ 60 years. Disability was assessed using the BI scale, with a score of 100 representing normal status, 65–95 as mild disability, 45–60 as moderate disability, and 0–40 as severe disability. The prevalence of disability was estimated by demographics and health characteristics, and their associations were explored by robust Poisson regression analysis.

**Results:**

The prevalence of disability among older adults was 19.4%, and the prevalence of mild, moderate, and severe disability was 16.8%, 1.5%, and 1.1%, respectively. All variables, including older age, residence in a rural area, higher number of hospitalizations, comorbidities, poor self-rated health, falls, cognitive impairment, mental impairment, and alienation from friends and relatives, were shown to be associated with a higher adjusted prevalence of disability. Only formal education can reduce the risk of disability.

**Conclusion:**

The prevalence of disability among older adults is high in both urban and rural settings in southwest China, and a number of important factors associated with disability have been identified. In addition to increased attention to the health status of older adults, further research on scientific management and effective disability interventions is needed.

**Supplementary Information:**

The online version contains supplementary material available at 10.1186/s12877-022-03193-2.

## Background

The term *disability* was first defined by Nagi in the 1960s [1]. The World Health Organization (WHO) has developed an updated framework [2] in 2002, which considers the term as an umbrella term for impairments, activity limitations and participation restrictions, and emphasizes that disability is a long-term interaction between the person and the overall environment in which the person lives. This implies that disability is a complex and multifactorial state involving multiple risk factors. Currently, there are no uniform standards and methods for assessing and classifying disability in older adults. In order to obtain comparable global health data, various tools for measuring disability have been developed [3–7]. The most commonly used one is the Barthel Index (BI) [8], which can evaluate the ability of older adults to perform daily living activities. It is characterized by its simplicity of operation, good reliability and sensitivity [9].

Human life expectancy has reached an all-time high and is on the rise [10], which will lead to the emergence of a large number of older adults with different degrees of disability [11]. Disability not only affects the quality of life and health outcomes of those people, but also significantly increases the cost of care [12]. Collecting data from representative studies in 37 countries, WHO reports that 14% of the 514 million older people (60 years and older) lack the basic skills to lead a meaningful and dignified life [10]. By the end of 2015, China's elderly population had reached 40.63 million, accounting for 18.3% of older adults in the same period [13]. It is conceivable that China, as the largest developing country globally, is facing a great socioeconomic burden.

Previous studies have explored a range of factors that may affect the physical functioning, activity, and social participation of older adults, including but not limited to sociodemographic characteristics, social networks, self-perceived health, cognitive functioning, mental health, disease burden, and repeat hospitalizations [14–18]. Since 2005, many similar studies have been conducted in most regions of China, but few studies have been conducted in the Southwest [19–22]. Given the regional differences in social and economic development, there are significant disparities in education, employment opportunities, social welfare, medical resources, and geriatric care services between Southwest and the eastern coastal regions of China. Sichuan Province, a representative province in southwest China, is characterized by a large rural population, a low level of education for the overall population, a multi-ethnic population, and uneven development across regions. Its GDP per capita is far below that of the eastern provinces [23]. In addition, data from the sixth national census released by China's National Bureau of Statistics in 2011 shows that Sichuan province has the largest population and the most severe aging problem in the southwest. This means that the disability of a large elderly population in this less developed part of the country has not received the attention it deserves. Although the local government has introduced some policies on care services for the disabled elderly, the overall disability situation in the province is not clear. Therefore, this study was conducted at a province-wide level to determine the prevalence and influencing factors of disability among the elderly in urban and rural areas of the province, so as to provide scientific references for local government interventions and the development of care policies.

From the above analysis, there are three gaps between this study and the previous ones. First, although disability among older adults is relatively common and has a significant impact on individuals, families, and society, relevant research is rare in the less developed regions of southwest China. Second, few large-scale studies specifically address disability among older adults in urban and rural communities. Finally, few studies on disability in the elderly encompass social factors, physical health, mental health, and cognitive function functioning.

## Method

### Participants

A cross-sectional survey was conducted in several districts/counties of Sichuan from March to September 2020. A total sample size of 16,536 older adults (age ≥ 60 years) was obtained through a multistage stratified random sampling procedure based on the latest resident population information in Sichuan, which was determined based on the following assumptions: prevalence = 20%, sampling error = 5.0%, CI = 95.0%, and non-response rate = 20.0%. The stratification in the sampling plan includes: 1) Based on the different characteristics of the age structure of the elderly in various cities and prefectures in Sichuan Province, the elderlies in various regional groups in the province are divided into four age groups: 60–69 years old, 70–79 years old, 80–89 years old and 90 years old and above; 2) Considering the characteristics of the population density distribution and economic zone development in Sichuan Province, the 21 prefectures in Sichuan Province are divided into six regional groups; 3) The third layer is districts/counties within each group in the second tier. We sampled the age groups and regional layers in equal proportions in turn, conducted systematic sampling in the third layer, and finally performed random sampling in the selected districts/counties. The sex ratio of subjects was controlled according to the sixth national census statistics.

### Data Collection

Physicians and nurses from local primary care facilities constituted the majority of the interviewers. They were asked to attend a comprehensive training course at the National Clinical Research Center for Geriatrics prior to the assessment, where they were trained in the use of Electronic Data Capture (EDC) to collect data through face-to-face interviews. Only those who passed both the theoretical and practical exams received a certificate of eligibility from the center. In addition, missing or incorrect questionnaires were reassessed. The entire assessment process of the survey was under the scrutiny of specialized personnel sent from their parent organization.

### Disability Measurement

The ADL was measured by the BI scale, which were essential in evaluating the independence of self-care among the aged [24], including personal hygiene, bathing self, feeding, toilet, stair climbing, dressing, bowel control, bladder control, ambulation, and chair/bed transfers. Its excellent reliability and validity values, as well as specific increments and values, have been detailed in previous studies [25]. The BI scale was used to assess disability, with scores of 0–40 representing severe impairment in activities of daily living, 45–60 representing moderate impairment, and 65–95 representing mild impairment.

### Characteristics

This study conceptualized the factors influencing disability into two areas: social factors and health status. Social factors included demographics such as age, gender, race, place of residence, marital status, education level, pre-retirement occupation, and consumption level. Health factors included hospitalization, chronic illness, self-rated health, accidents, cognitive status, and mental health. The hospitalization rate in the previous year, chronic disease, self-rated health and accidents within one month were recorded based on the subjects' reports. Comorbidity was defined as the presence of two or more chronic conditions at the same time. Based on the subjects' assessments, their self-rated health status was classified into three categories, including poor, general, and good.

The cognitive state was measured using the Mini-cog test widely used with high reliability and validity [26]. The test consisted of a 3-word recall and clock drawing test (CDT). Subjects who recalled 0 words were classified as "dementia", those who recalled 1–2 words were classified according to the CDT (abnormal = "MCI", normal = "normal"), and those who recalled 3 words were classified as "normal".

Anxiety symptoms were screened by GAD-2 [27], with subjects with a score ≥ 3 being regarded as anxious, and depressive symptoms were evaluated by PHQ-2 [28], with subjects with a score ≥ 3 being regarded as depressed. Subjects with both depressive and anxiety symptoms were assigned to the "comorbid depressive and anxiety symptoms" group, those with anxiety symptoms alone to the "Anxiety" group, those with depressive symptoms alone to the "Depression" group, and those with none of the above symptoms to the "normal" group.

Family and friend networks were assessed using LSNS-6 [29], which was highly reliable and valid and has been used in many countries [30]. This measurement involves six questions: three for friend ties and three for family ties. Each question was scored on a scale of 0 to 5, with scores ranging from 0 to 30. "Social isolation" was considered for a total score of < 12. When the total scores ≥ 12, subjects with a score of < 6 for the friend part were classified as "alienation from friends", and those with a score of < 6 for the family part as "alienation from relatives".

### Statistical Analysis

Continuous variables were described as mean ± SD, and categorical variables were described as percentages (*n*, %). T-test and X^2^ test were adopted to compare the differences in demographic and health characteristics between groups, and logistic regression models were performed to correct for relevant confounders. We chose a robust Poisson regression model for the analysis [31, 32]. The specific model assumptions can be found in Supplemental Files (S1). Robust Poisson random effects models can be implemented through GLMs (link function: log, error distribution: Poisson) in Stata 15.1. The multivariate model with minimum Akaike Information Criteria (AIC) and Bayesian Information Criteria (BIC) was considered the best fitting model. All analyses were performed with Stata 15.1, and  < 0.05 (two-sided) were considered statistically significant.

## Results

### Prevalence and distribution of disability

**Fig. 1:**
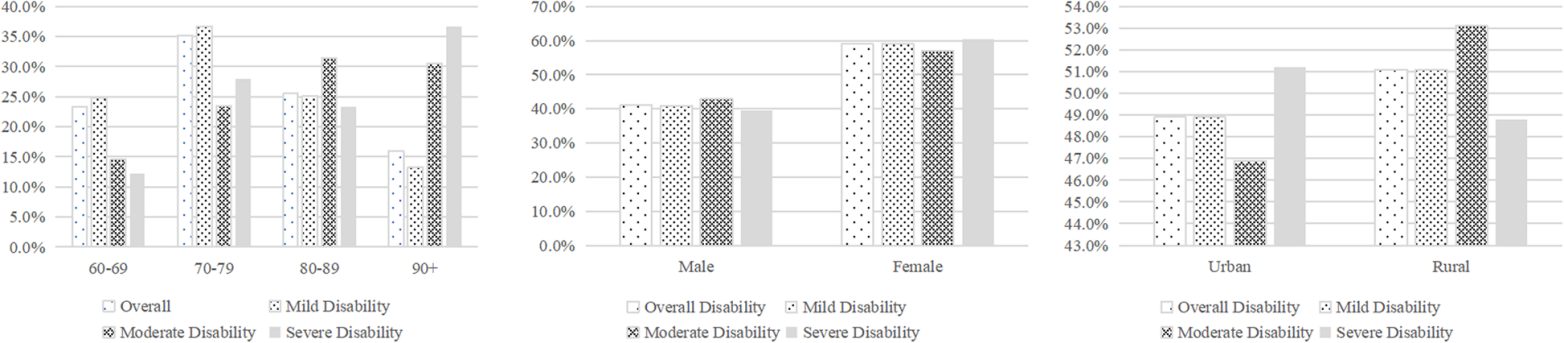
A combination of three figures. Disability was divided into three levels: mild, moderate and severe. Age was divided into four groups. The one on the left showed the distribution of different disabilities by age. The middle one showed the distribution of different disabilities by genders. The one on the right showed the distribution of different disabilities by residency areas

A total of 15,385 responses were collected, including 8368(54.4%) from female respondents. In this study, 19.4% of the subjects experienced disability, and the incidence of mild, moderate, and severe disability was 16.8%, 1.5%, and 1.1%, respectively. Significant differences were found in the distribution of disability by age group, gender, and area of residence (S2, Fig. 1). Figure 1 shows that the older people are, the higher the prevalence of disability and the degree of impairment. The prevalence of the three types of disabilities increased sharply among people aged 70–79 years, and the proportion of people with severe disabilities reached 36.6% among those aged 90 years or older. Among those with severe disabilities, the gender gap was the largest, with women accounting for 60.5%. In addition, we found that 51.1% of older adults with disabilities were from rural areas, more than 50% of older adults with mild and moderate disabilities were from rural areas, while only 48.8% of older adults with severe disabilities were from rural areas.

### The social relevance of disability

In the present study, the mean age of the disabled group (77.7 ± 9.5 years) was greater than that of the non-disabled group (70.7 ± 6.8 years). As shown in Table 1, there was a statistically significant difference between the different age groups (*P* < 0.001). It was found that people with disabilities were more likely to be older women and people living in rural areas or former farmers (*P *< 0.001). In addition, disability was more common among older adults who were widowed, had no formal education, or had low consumption levels (*P* < 0.001). The results also showed that alienation from relatives and friends was significantly associated with disability (*P* < 0.001).

**Table 1:** Distribution of disability by demographics characteristics

**Demographic Characteristics**	**Total sample *N* = 15,385**	**Without disability *N* = 12,404**	**With disability *N* = 2981**	**χ**^2^*	***P*-value**
**Age(years)**					
60–69	7009(45.6%)	6314 (50.9%)	695 (23.3%)	2100.0	< 0.001
70–79	5757(37.4%)	4707 (37.9%)	1050 (35.2%)		
80–89	1969(12.8%)	1209 (9.7%)	760 (25.5%)		
≥ 90	650(4.2%)	174 (1.4%)	476 (16.0%)		
**Gender**					
Male	7017(45.6%)	5795 (46.7%)	1222 (41.0%)	31.8	< 0.001
Female	8368(54.4%)	6609 (53.3%)	1759 (59.0%)		
**Residency area**					
Urban	8534(55.5%)	7077 (57.1%)	1457 (48.9%)	65.1	< 0.001
Rural	6851(44.5%)	5327 (42.9%)	1524 (51.1%)		
**Marital status**					
Married	11,398(74.1%)	9626 (77.6%)	1772 (59.4%)	463.1	< 0.001
Widowed	3617(23.5%)	2470 (19.9%)	1147 (38.5%)		
Divorced	177(1.2%)	153 (1.2%)	24 (0.8%)		
Never married	193(1.3%)	155 (1.2%)	38 (1.3%)		
**Education levels**					
No formal education	3247(21.1%)	2195 (17.7%)	1052 (35.3%)	498.7	< 0.001
Primary school	7702(50.1%)	6329 (51.0%)	1373 (46.1%)		
Secondary school	3176(20.6%)	2792 (22.5%)	384 (12.9%)		
High school and over	1060(8.2%)	1088 (8.8%)	172 (5.8%)		
**Occupation**					
Farmer	10,940(71.1%)	8667 (69.9%)	2273 (76.2%)	48.2	< 0.001
worker	1573(10.2%)	1314 (10.6%)	259 (8.7%)		
Office staff	1961(12.8%)	1659 (13.4%)	302 (10.1%)		
Other	911(5.9%)	764 (6.2%)	147 (4.9%)		
**Consumption level(yuan/month)**					
< 500	3513(22.8%)	2639 (21.3%)	874 (29.3%)	97.6	< 0.001
500–1000	5505(35.8%)	4527 (36.5%)	978 (32.8%)		
1000–1500	3187(20.7%)	2670 (21.5%)	517 (17.3%)		
> 1500	3180(20.7%)	2568 (20.7%)	612 (20.5%)		
**Social networks**					
Normal	7406(48.1%)	6358 (51.3%)	1048 (35.2%)	276.5	< 0.001
Alienation from friends	3069(20.0%)	2340 (18.9%)	729 (24.5%)		
Alienation from relatives	695(4.5%)	574 (4.6%)	121 (4.1%)		
Social isolation	4215(27.4%)	3132 (25.2%)	1083 (36.3%)		

^*^:Value of chi-square test for comparing across categories for categorical variables

### Health-related factors of disability

As shown in Table 2, the distribution of disability by health characteristics showed a correlation between disability and hospitalization rates/days (*P* < 0.001). Disability was more common in patients with chronic conditions, and its incidence increased with the number of comorbid conditions (*P* < 0.001). As expected, the number of disabilities increased with worsening self-rated health status and the incidence of falls (*P* < 0.001). In addition, impairment in cognitive function and the occurrence of depressive and anxiety symptoms were significantly associated with the incidence of disability (*P* < 0.001).

**Table 2:** Distribution of disability by health Characteristics

Health Characteristics	Total sample *N* = 15,385	Without disability *N *= 12,404	With disability *N *= 2981	χ^2^*	*P*-value
**Hospitalization times(in one year)**					
never	12,013(78.1%)	10,009 (80.7%)	2004 (67.2%)	328.9	< 0.001
= 1	2238(14.6%)	1683 (13.6%)	555 (18.6%)		
≥ 2	1134(7.37%)	712 (5.7%)	422 (14.2%)		
**Hospitalization days(in one year)**					
< 7	436(12.9%)	346 (14.4%)	90 (9.2%)	16.9	< 0.001
≥ 7	2936(87.1%)	2049 (85.6%)	887 (90.8%)		
**Chronic diseases numbers**					
0	5786(37.6%)	5024 (40.5%)	762 (25.6%)	341.0	< 0.001
1	3525(22.9%)	2908 (23.4%)	617 (20.7%)		
≥ 2 (Co-morbidity)	6074(39.5%)	4472 (36.1%)	1602 (53.7%)		
**Self-rated health**					
Good	4196(27.3%)	3880 (31.3%)	316 (10.6%)	1300.0	< 0.001
General	8410(54.7%)	6925 (55.8%)	1485 (49.8%)		
Poor	2779(18.1%)	1599 (12.9%)	1180 (39.6%)		
**Falls within 30 days**					
No	14,695(95.5%)	11,975 (96.5%)	2720 (91.2%)	157.4	< 0.001
Yes	690(4.5%)	429 (3.5%)	261 (8.8%)		
**Cognitive status**					
Normal	8142(52.9%)	7187 (57.9%)	955 (32.0%)	1300.0	< 0.001
Mild impairment	6064(39.4%)	4673 (37.7%)	1391 (46.7%)		
Dementia	1179(7.7%)	544 (4.4%)	635 (21.3%)		
**Mental disorders**					
Normal	14,141(91.9%)	11,740 (94.6%)	2401 (80.5%)	674.6	< 0.001
Anxiety	239(1.6%)	153 (1.2%)	86 (2.9%)		
Depression	459(3.0%)	253 (2.0%)	206 (6.9%)		
Comorbid^**^	546(3.6%)	258 (2.1%)	288 (9.7%)		

^*^:Value of chi-square test for comparing across categories for categorical variables

^*^^*^: Comorbid = Comorbid depressive and anxiety symptoms

### Distributions of ADL Component

**Fig. 2:**
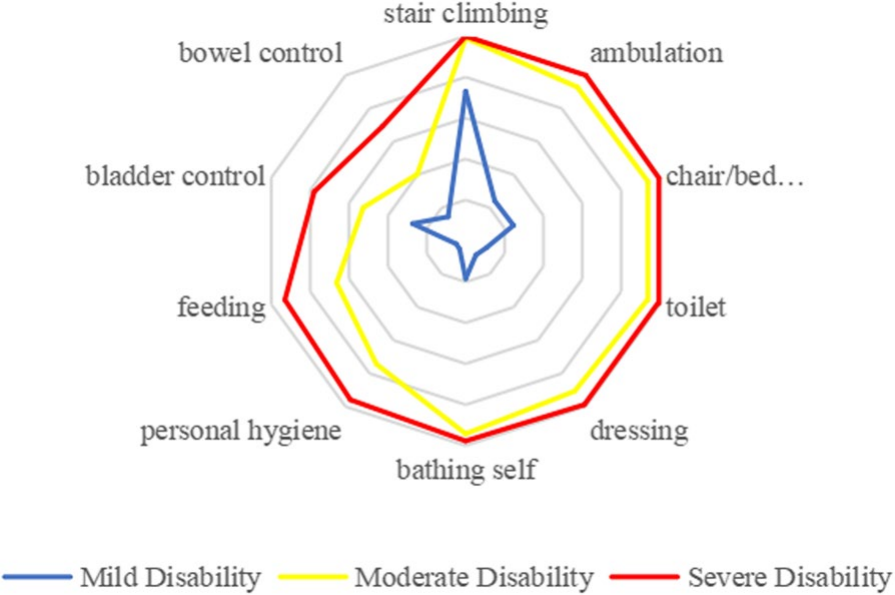
Distributions of ADL disability in different items. The radar chart showed us the rate of impairment of 10 competencies in the BI of the older adults at each level of disability

**Fig. 3:**
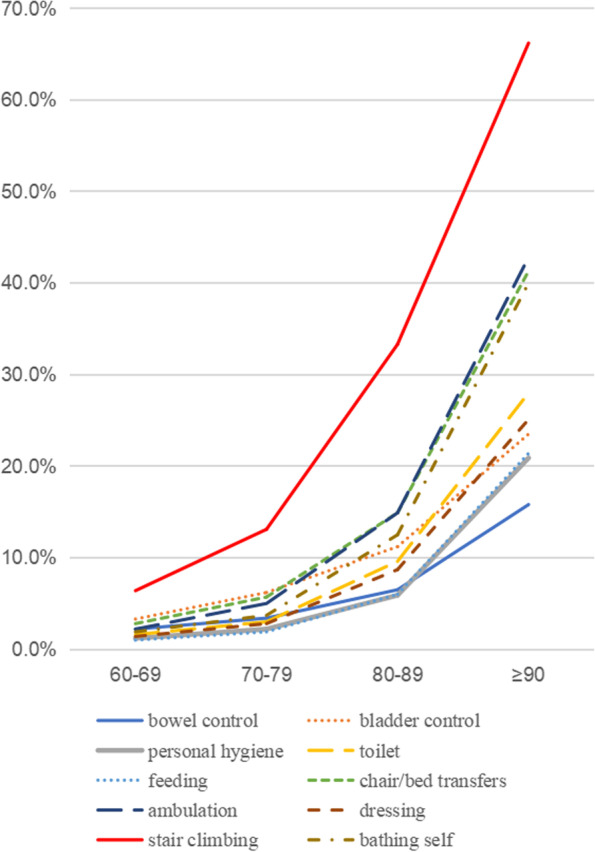
Distribution of different ability-impaired people in different age groups. It showed us the rate of impairment of 10 competencies in the BI of the older adults at different age groups

Table 3 and Fig. 2 show the distribution of impairments for the different items in the BI scale. Functional impairments in older adults with mild disabilities mainly included stair climbing (73.1%), bladder control (27.3%), and chair/bed transfer (24.6%). Those with moderate to severe disabilities showed similarities in limitations in abilities, mainly involving lower limb function. Figure 3 confirms that the incidence of impairments in all daily activities under assessment saw a significant increase with aging, among which the impairment of climbing was the earliest and the most common one, followed by ambulation, chair/bed transfers, and bathing, at a generally consistent pace.

**Table 3:** Prevalence of impairment for individual items in different groups

Items	Mild Disability	Moderate Disability	Severe Disability
stair climbing	73.0%	99.1%	100.0%
ambulation	24.2%	92.9%	100.0%
chair/bed transfers	24.6%	93.8%	99.4%
toilet	10.7%	93.4%	98.8%
dressing	8.5%	90.7%	98.8%
bathing self	18.4%	94.3%	97.7%
personal hygiene	5.0%	74.3%	95.9%
feeding	4.8%	66.4%	93.0%
bladder control	27.3%	52.7%	77.9%
bowel control	14.5%	40.3%	69.2%

### Factors Associated with Disability

**Fig. 4:**
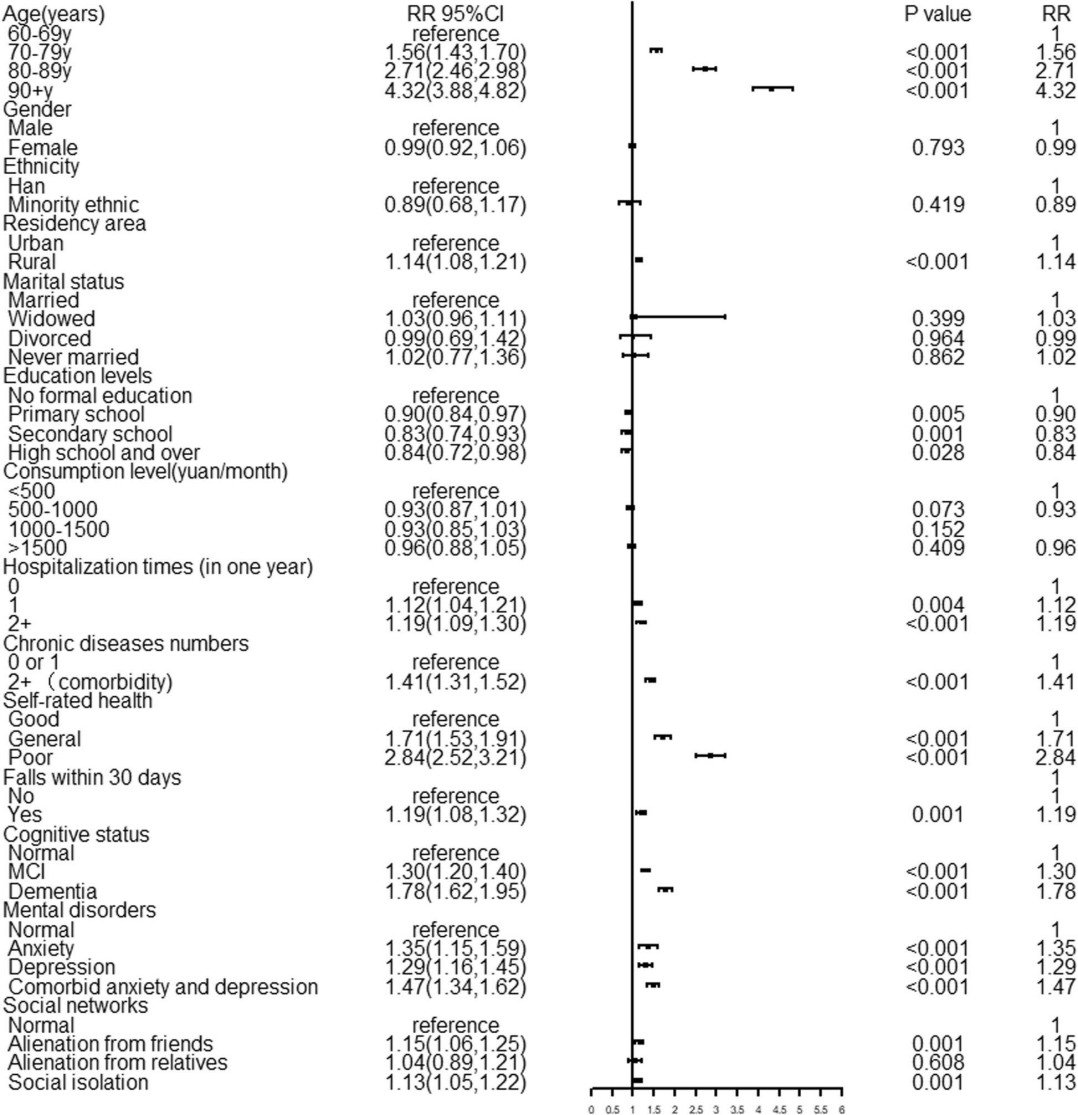
Factors associated with disability. Adjusted rate ratio were calculated using robust Poisson regression. Adjusted for all variables. Abbreviation: CI = confidence interval, MCI = mild cognitive impairment

Based on the results of many correlation studies and univariate analysis, we finally included 14 relevant variables to construct different models [16, 18, 33–36]. The best fitting model had an AIC value of 0.88, a BIC value of -140,574.20. After adjusting for all significant variables (S3, Fig. 4), older age (RR = 4.32; 3.88–4.82), living in rural areas (RR = 1.14; 1.08–1.21), increased hospitalization (RR = 1.19; 1.09–1.30), co-morbidity (RR = 1.41; 1.31–1.52), self-rated poor health ( RR = 2.84; 2.52–3.21), recent falls (RR = 1.19; 1.08–1.32), MCI (RR = 1.30; 1.20–1.40), dementia (RR = 1.78; 1.62–1.95), anxiety (RR = 1.35; 1.15–1.59), depression (RR = 1.29; 1.16–1.45), comorbid depressive and anxiety symptoms (RR = 1. 47; 1.34–1.62), alienation from friends (RR = 1.15; 1.06–1.25), and social isolation (RR = 1.13; 1.05–1.22) increased the risk of disability in older adults (*P* < 0.05). Only higher levels of education could be used as a protective factor for disability. In the fully adjusted model, gender, marital status and consumption level were no longer associated with the prevalence of disability (*P* > 0.05).

## Discussion

In this study, the prevalence of disability among older adults in Sichuan Province was 19.4%, higher than the worldwide average [37], as well as the results in the reports of many countries such as Poland [38] and some domestic studies [39]. Such a high disability rate among the elderly in a place with low per capita income, severe aging, a significant exodus of the working population, uneven regional development, and limited medical resources undoubtedly poses a considerable challenge to the local health care system [23, 40]. The key to solving this problem lies in reliable estimation and effective disability prevention. Therefore, this study aimed to investigate the prevalence and influencing factors of disability among the elderly in urban and rural Sichuan, which is of great significance. To the best of our knowledge, this is the first large-scale survey of disability in urban and rural areas in southwest China. Theoretically, these results can be generalized to Southwest China and other populations with similar characteristics.

Similar to the findings of other scholars [34], the earliest and most severe impairments in the disabled elderly involved their lower limb function, such as stair climbing, ambulation, and bathing self. The worsened lower limb function can be explained by related factors. 1) Natural aging reduces lower limb muscle strength and quality [41]; 2) Chronic diseases, such as osteoarthritis [42], diabetes, and chronic obstructive pulmonary disease [43], and falls will cause mobility limitation, further decreasing their physical function; 3) Dementia will cause gait disorders, decreased balance function, and increased risk of falls, eventually increasing physical dependence [44]; 4) In the case of repeated hospitalization [45], physical inactivity in the elderly, prolonged bed rest, and reduced intake all decrease lower limb function. Therefore, it is crucial to maintain lower limb mobility in the elderly and promote post-injury rehabilitation. Health education and targeted physical activity interventions have been introduced in many countries [46]. However, the rapid aging of the population continues to leave low- and middle-income countries unprepared. In 2016, the Chinese government included "healthy aging" in its national development program and introduced long-term care insurance for the elderly with disabilities in many pilot cities [47]. This insurance focuses on financial compensation, while care services are mostly provided by third-party institutions. The quality and quantity of daily care services still do not meet the existing needs of these elderly people. 39.6% of elderly people with disabilities self-rated their health as poor, which may be related to unmet needs [48]. What is certain is that the burden of care for moderate and severe disabilities is high. We need to reduce the future burden of care by working to prevent mild disabilities, especially in economically disadvantaged areas [49]. By developing appropriate physical activity patterns, improving the quality of chronic disease management, and optimizing inpatient management concepts, we can maximize the prevention of lower extremity mobility loss in older adults.

The results of this study suggest that aging contributes to the onset and progression of disability, as has been confirmed in numerous articles [50]. However, follow-up cohort studies are still needed to elucidate the specific trajectory of disability. Aging poses a number of psychiatric problems in older adults that are caused by many factors, including: 1) decreased sensory function [51]; 2) decreased adaptability to environmental changes and social roles and status; and 3) increased likelihood of exposure to negative life events, such as retirement and death of a relative. In this study, 8.2% of the elderly screened positive for mental disorders, and the rates of positive depression and anxiety-depression comorbidity were significantly higher in the disabled elderly. In addition, less interaction with family and friends increased the incidence of disability. However, alienation from family alone did not have a statistically significant effect on disability, which may be related to the current situation of empty nesters in China. Older adults rely more on interactions with friends than the next generation who are busy with work. According to the meta-analysis, the prevalence of depression among Chinese empty nesters was 38.6% [52]. Research suggests a bidirectional relationship between social isolation and depression or anxiety, which naturally accelerates the onset of disability [53]. However, public spending on mental health in developing countries remains low and is mostly focused on psychiatric hospitals [54]; while, these hospitals do not provide community mental health services that can provide long-term care and support. As early as 2004, China attempted to establish a comprehensive community mental health system; however, the system continues to face significant challenges due to low national awareness of the need for mental health services, lack of specialized physicians, and financial difficulties [55]. This study will provide a reference for regional epidemiological data on mental disorders in the elderly, and also call for relevant authorities to pay attention to the mental health problems of the elderly.

Consistent with previous studies [56], our findings suggest a strong association between cognitive function and disability. The survey showed that 1,179 elderly people had positive dementia screening results, and the proportion of dementia in the disabled population was 4.8 times higher than that in the non-disabled population, which fully indicates that dementia is an important cause of loss of self-care in the elderly. As the country with the largest number of dementia patients in the world [57], China has not yet established a service system specifically for dementia. Early detection of cognitive impairment is not possible due to low public awareness of dementia and the lack of routine screening mechanisms in most medical facilities. Many people with dementia rely on home care, which is increasingly being incorporated into long-term care. However, the lack of caregivers with spiritual and professional background makes it difficult to improve the quality of life of these elderly [58]. Therefore, the following steps should focus on increasing public awareness of dementia, earlier identification and prevention of cognitive impairment, and the establishment of a joint disability service system for dementia.

Statistically significant differences in disability were observed between genders, and the older women were more prone to experience functional impairment. However, after adjusting for other variables, the incidence of disability became similar between genders following adjustment of other variables, which might be explained by higher rates of chronic diseases such as osteoarthritis, dementia, falls, and mental disorder in women [59], as well as differences in body composition and life expectancy gaps [60]. For social factors, the analysis showed that older adults who live in urban areas and have formal education are better able to maintain their abilities, as has been well documented in many studies. This may be because they have access to health knowledge and resources from a variety of sources [61] as well as a range of social and recreational activities, which can help them maintain good mental health [62]. Despite China's increasing spending on healthcare and health, inefficiencies and uneven distribution of resources persist. Because of the differences in economics, healthcare, urbanization, and population density between the east and west, it is difficult to see a boom in care services and elderly care in the rural west anytime soon [63]. This study will offer policy implications and help local elder population to improve the quality of life.

This study has several strengths. First, this large sample study can provide high-quality and rich information for existing disability research, and the findings are important for follow-up studies and the global literature on the disability process and its associated factors. Second, comprehensive training of professional bodies and various quality control measures yielded reliable data. Finally, the study results provide a scientific basis for government policies and resource allocation to enable local older adults to have a higher quality of life and gradually achieve healthy aging. Several limitations should be taken into account when interpreting this study. As this study is a cross-sectional study, the results failed to determine a causal relationship between influencing factors and disability. Therefore, although many factors influencing disability were identified in this study, it cannot be denied that disability may also influence related factors to some extent. Further cohort studies are needed to determine the causal relationships. Some of the data on health characteristics and ADL items were self-reported, and biases in recall and reporting may have affected the information. Future studies should add more objective indicators and try to assess disability in multiple dimensions, rather than just selecting ADL as the only criterion for determining disability. This study served as a baseline investigation for our project, and more follow-up and exploration of interventions are needed in the future.

## Conclusions

A higher prevalence of disability was found among urban and rural older adults in Sichuan, where disability was strongly associated with aging, lower education levels, living in rural areas, hospitalization, co-morbidities, self-rated poor health, falls, cognitive impairment, psychological problems, and changes in social networks. The findings underscore the need for early screening for disability, effective prevention policies, smaller urban–rural disparities, and age-friendly society.

## Supplementary Information

**Additional file 1.**

## Data Availability

The datasets generated and analysed during the current study are not publicly available due to this is a newly database which are confidential and the authors do not have permission to share data. But this dataset is also available from the corresponding author on a reasonable request.

## Abbreviations

ADL: Activities of Daily Living
WHO: The World Health Organization
EDC: Electronic Data Capture
BI: The Barthel Index
MCI: Mild cognitive impairment
GAD-2: The 2-item Generalized Anxiety Disorder scale
PHQ-2: The 2-item Patient Health Questionnaire depression module
LSNS-6: The abbreviated Lubben Social Network Scale
SD: Standard deviation
CI: Confidence interval
