# Supplementary material for: Prevalence and correlates of disability among urban–rural older adults in Southwest China: a large, population-based study
Source: BMC Geriatr. 2022 Jun 24;22:517. doi: 10.1186/s12877-022-03193-2 (PMC9229854; doi:10.1186/s12877-022-03193-2)
Supplement: Supplementary file 1 — Additional file 1. [file 12877_2022_3193_MOESM1_ESM.docx]

**Prevalence and correlates of disability among urban-rural older adults in Southwest China: a large, population-based study**

Runjuan Qiao^1^, Shuli Jia^2^, Wanyu Zhao^2^, Xin Xia^2^, Qiaoli Su^1^, Lisha Hou^2^, Daiping Li^2^, Fengjuan Hu^2^, Birong Dong^1,2^

^1^National Clinical Research Center for Geriatrics and Department of General Practice, West China Hospital, Sichuan University, Chengdu, China.

^2^Center of Gerontology and Geriatrics, West China Hospital, Sichuan University, Chengdu, China.

**Corresponding author**

Dr. Birong Dong^1,2^

1 National Clinical Research Center for Geriatrics, West China Hospital, Sichuan University, GuoXueXiang 37, Chengdu, China, 610041.

2 Center of Gerontology and Geriatrics, West China Hospital, Sichuan University, GuoXueXiang 37, Chengdu, China, 610041.

E-mail address: Birongdong123@outlook.com

Fax: +86-028-85421550

Tel: +86-028-85421550

**Supplemental Files**

**S1. Random effects model for robust Poisson regression:**

First, let $y_{ki}$ and $X_{ki}=(x_{1ki},x_{2ki},...,x_{pki})^{T}$ be the binary outcome variable of the i-th$(i=1,2,\ldots,n_{k})$ individual in the k-th$(k=1,2,\ldots,K)$ class and the $P*1$ dimensional explanatory variable vector, respectively, and let $u_{k}$ be the random effect of the k-th class. Then, construct the random effect model:

$$\log(p_{ki})=\beta_{0}+\sum_{p=1}^{P} \beta_{p}x_{pki}+u_{k}$$

where $p_{ki}=\Pr(y_{ki}=1|X_{ki})$. Moreover, the connection function adopts the log function, $\beta_{p}$ is the fixed effect parameter, and reflects the effect of the fixed effect $x_{p}$ on the logarithm of the outcome probability, and $RR(PR)=\exp(\beta_{p})$; assuming that the random effect $u_{k}$ comes from the normal distribution population, that is:

$$u_{k}\sim N(0,\sigma_{u}^{2})$$

The random effects variance $\sigma_{u}^{2}$ reflects the degree to which the outcome variable is clustered within the class.

Then, Huber's robust "sandwich" variance is introduced to construct a robust Poisson random effects model:

$$Var(\beta)=A^{-1}BA^{-1}$$

where $A=\sum_{i=1}^{n} X_{i}X_{i}^{T}\text{p}_{i} B=\sum_{i=1}^{n} X_{i}(y_{i}-p_{i})^{2}X_{i}^{T}$.

**S2.**The prevalence of ADL disability in different age, gender, areas groups.

| **Age(years)** | **With**  **Disability** | **Mild**  **Disability** | **Moderate**  **Disability** | **Severe**  **Disability** |
| --- | --- | --- | --- | --- |
| Overall | 19.4% | 16.8% | 1.5% | 1.1% |
| Age（years） |  |  |  |  |
| 60-69 | 10.0% | 9.2% | 0.5% | 0.3% |
| 70-79 | 18.2% | 16.5% | 0.9% | 0.8% |
| 80-89 | 38.6% | 33.0% | 3.6% | 2.0% |
| ≥90 | 73.2% | 52.9% | 10.6% | 9.7% |
| Gender |  |  |  |  |
| Male | 41.0% | 40.9% | 42.9% | 39.5% |
| Female | 59.0% | 59.1% | 57.1% | 60.5% |
| Residency area |  |  |  |  |
| Urban | 48.9% | 48.9% | 46.9% | 51.2% |
| Rural | 51.1% | 51.1% | 53.1% | 48.8% |

**S3.** Robust Poisson Regression Model Analysis of influencing factors of disability

|  | **RR** | **Robust SE** | **95%CI** | ***p*-value** |
| --- | --- | --- | --- | --- |
| Age（years） |  |  |  |  |
| 60-69 | 1 | / | / | / |
| 70-79 | 1.56 | 0.07 | 1.43-1.70 | <0.001 |
| 80-89 | 2.71 | 0.13 | 2.46-2.98 | <0.001 |
| ≥ 90 | 4.32 | 0.24 | 3.88-4.82 | <0.001 |
| Gender |  |  |  |  |
| Male | 1 | / | / | / |
| Female | 0.99 | 0.03 | 0.92-1.06 | 0.793 |
| Race |  |  |  |  |
| Han | 1 | / | / | / |
| Ethnics | 0.89 | 0.12 | 0.68-1.17 | 0.419 |
| Residency area |  |  |  |  |
| Urban | 1 | / | / | / |
| Rural | 1.14 | 0.03 | 1.08-1.21 | <0.001 |
| Marital status |  |  |  |  |
| Married | 1 | / | / | / |
| Widowed | 1.03 | 0.04 | 0.96-1.11 | 0.399 |
| Divorced | 0.99 | 0.18 | 0.69-1.42 | 0.964 |
| Never married | 1.02 | 0.15 | 0.77-1.36 | 0.862 |
| Education levels |  |  |  |  |
| No formal education | 1 | / | / | / |
| Primary school | 0.9 | 0.03 | 0.84-0.97 | 0.005 |
| Secondary school | 0.83 | 0.05 | 0.74-0.93 | 0.001 |
| High school and over | 0.84 | 0.07 | 0.72-0.98 | 0.028 |
| Consumption level(yuan/month) | | | | |
| ＜500 | 1 | / | / | / |
| 500-1000 | 0.93 | 0.04 | 0.87-1.01 | 0.073 |
| 1000-1500 | 0.93 | 0.04 | 0.85-1.03 | 0.152 |
| ＞1500 | 0.96 | 0.04 | 0.88-1.05 | 0.409 |
| Hospitalization times（in one year） | | | | |
| never | 1 | / | / | / |
| =1 | 1.12 | 0.05 | 1.04-1.21 | 0.004 |
| ≥ 2(Co-morbidity) | 1.19 | 0.05 | 1.09-1.30 | <0.001 |
| Chronic diseases numbers |  |  |  |  |
| 0 | 1 | / | / | / |
| 1 | 1.14 | 0.05 | 1.05-1.25 | 0.003 |
| ≥ 2 | 1.41 | 0.06 | 1.31-1.52 | <0.001 |
| Self-rated health |  |  |  |  |
| Good | 1 | / | / | / |
| General | 1.71 | 0.1 | 1.53-1.91 | <0.001 |
| Poor | 2.84 | 0.18 | 2.52-3.21 | <0.001 |
| Falls within 30 days |  |  |  |  |
| No | 1 | / | / | / |
| Yes | 1.19 | 0.06 | 1.08-1.32 | 0.001 |
| Cognitive status |  |  |  |  |
| Normal | 1 | / | / | / |
| MCI | 1.3 | 0.05 | 1.20-1.40 | <0.001 |
| Dementia | 1.78 | 0.08 | 1.62-1.95 | <0.001 |
| Mental disorders |  |  |  |  |
| Normal | 1 | / | / | / |
| Anxiety | 1.35 | 0.11 | 1.15-1.59 | <0.001 |
| Depression | 1.29 | 0.07 | 1.16-1.45 | <0.001 |
| Comorbid | 1.47 | 0.07 | 1.34-1.62 | <0.001 |
| Social networks |  |  |  |  |
| Normal | 1 | / | / | / |
| Alienation from friends | 1.15 | 0.05 | 1.06-1.25 | 0.001 |
| Alienation from relatives | 1.04 | 0.08 | 0.89-1.21 | 0.608 |
| Social isolation | 1.13 | 0.04 | 1.05-1.22 | 0.001 |

**Abbreviation:** RR = rate ratio, Robust SE = robust standard error, CI = confidence interval, MCI= mild cognitive impairment. Adjusted for all significant variables.
